# Hitchhiking to Speciation

**DOI:** 10.1371/journal.pbio.1001498

**Published:** 2013-02-26

**Authors:** Daven C. Presgraves

**Affiliations:** Department of Biology, University of Rochester, Rochester, New York, United States of America

## Abstract

The modern evolutionary synthesis codified the idea that species exist as distinct entities because intrinsic reproductive barriers prevent them from merging together. Understanding the origin of species therefore requires understanding the evolution and genetics of reproductive barriers between species. In most cases, speciation is an accident that happens as different populations adapt to different environments and, incidentally, come to differ in ways that render them reproductively incompatible. As with other reproductive barriers, the evolution and genetics of interspecific hybrid sterility and lethality were once also thought to evolve as pleiotripic side effects of adaptation. Recent work on the molecular genetics of speciation has raised an altogether different possibility—the genes that cause hybrid sterility and lethality often come to differ between species not because of adaptation to the external ecological environment but because of internal evolutionary arms races between selfish genetic elements and the genes of the host genome. Arguably one of the best examples supporting a role of ecological adaptation comes from a population of yellow monkey flowers, *Mimulus guttatus*, in Copperopolis, California, which recently evolved tolerance to soil contaminants from copper mines and simultaneously, as an incidental by-product, hybrid lethality in crosses with some off-mine populations. However, in new work, Wright and colleagues show that hybrid lethality is not a pleiotropic consequence of copper tolerance. Rather, the genetic factor causing hybrid lethality is tightly linked to copper tolerance and spread to fixation in Copperopolis by genetic hitchhiking.

New species arise when populations gradually evolve intrinsic reproductive barriers to interbreeding with other populations [1]–[3]. Two species can be reproductively isolated from one another in ways that prevent the formation of interspecific hybrids—the species may, for instance, have incompatible courtship signals or occupy different ecological habitats. Two species can also be reproductively isolated from one another if interspecific hybrids are formed but are somehow unfit—the hybrids may be sterile, inviable, or may simply fall between parental ecological niches. All forms of reproductive isolation limit the genetic exchange between species, preventing their fusion and facilitating their further divergence. Understanding the genetic and evolutionary basis of speciation—a major cause of biodiversity—therefore involves understanding the genetics and evolutionary basis of the traits that mediate reproductive isolation.

Most reproductive barriers arise as incidental by-products of selection—either ecological adaptation or sexual selection. For these cases, the genetic basis of speciation is, effectively, the genetics of adaptation. But hybrid sterility and lethality have historically posed two special problems. Darwin [4] devoted an entire chapter of his *Origin of Species* to the first problem: as the sterility or lethality of hybrids provides no advantage to parents, how could the genetic factors involved possibly evolve by natural selection? The second problem was recognized much later [5], after the rediscovery of Mendelian genetics: if two species (with genotypes *AA* and *aa*) produce, say, sterile hybrids (*Aa*) due to an incompatibility between the *A* and *a* alleles, then how could, e.g., the *AA* genotype have evolved from an *aa* ancestor in the first place without passing through a sterile intermediate genotype (*Aa*)? Not only does natural selection not directly favor the evolution of hybrid sterility or lethality, but there is reason to believe natural selection positively prevents its evolution.

Together these problems stymied evolutionists and geneticists for decades. T.H. Huxley [6] and William Bateson [5], writing decades apart, each branded the evolution of hybrid sterility one of the most serious challenges for a then-young evolutionary theory. Darwin had, in fact, offered a simple solution to the first problem. Namely, hybrid sterility and lethality are not advantageous per se but rather “incidental on other acquired differences" [4]. Then Bateson [5], in a few short, forgotten lines solved the second problem (see [7]). Later, Dobzhansky [2] and Muller [8] would arrive at the same solution, showing that hybrid sterility or lethality could evolve readily, unopposed by natural selection, under a two-locus model with epistasis. In particular, they imagined that separate populations diverge from a common ancestor (genotype *aabb*), with the *A* allele becoming established in one population (*AAbb*) and the *B* allele in the other (*aaBB*); while *A* and *B* alleles must function on their respective genetic backgrounds, there is no guarantee that the *A* and *B* alleles will be functionally compatible with one another. Hybrid sterility and lethality most likely result from incompatible complementary genetic factors that disrupt development when brought together in a common hybrid genome. Dobzhansky [2] and Muller [8] could point to a few supporting data in fish, flies, and plants. Notably, like Darwin, neither speculated on the forces responsible for the evolution of the genetic factors involved.

Today, there is no doubt that the Dobzhansky-Muller model is correct, as the data for incompatible complementary genetic factors is now overwhelming [1],[9]. In the last decade, a fast-growing number of speciation genes involved in these genetic incompatibilities have been identified in mice, fish, flies, yeast, and plants [9]–[11]. Perhaps not surprisingly, these speciation genes often have histories of recurrent, adaptive protein-coding sequence evolution [10],[11]. The signature of selection at speciation genes has been taken by some as tacit evidence for the pervasive role of ecological adaptation in speciation, including the evolution of hybrid sterility and lethality [12]. What is surprising, however, from the modern molecular analysis of speciation genes is how often their rapid sequence evolution and functional divergence seems to have little to do with adaptation to external ecological circumstances. Instead, speciation genes often (but not always [9]–[11]) seem to evolve as by-products of evolutionary arms races between selfish genetic elements—e.g., satellite DNAs [13],[14], meiotic drive elements [15], cytoplasmic male sterility factors [16]—and the host genes that regulate or suppress them [9]–[11],[17]. The notion that selfish genes are exotic curiosities is now giving way to a realization that selfish genes are common and diverse, each generation probing for transmission advantages at the expense of their bearers, fueling evolutionary arms races and, not infrequently, contributing to the genetic divergence that drives speciation. Indeed, the case has become so strong that examples of hybrid sterility and lethality genes that have evolved in response to ecological challenges (other than pathogens) appear to be the exception [9],[11],[17].

Perhaps the most clear-cut case in which a genetic incompatibility seems to have evolved as a by-product of ecological adaptation comes from populations of the yellow monkey flower, *Mimulus guttatus*, from Copperopolis (California, U.S.A.). In the last ∼150 years, the Copperopolis population has evolved tolerance to the tailings of local copper mines (Figure 1). These copper-tolerant *M. guttatus* plants also happen to be partially reproductively isolated from many off-mine *M. guttatus* plants, producing hybrids that suffer tissue necrosis and death. In classic work, Macnair and Christie showed that copper tolerance is controlled by a single major factor [18] and hybrid lethality, as expected under the Dobzhansky-Muller model, by complementary factors [19]. Surprisingly, in crosses between tolerant and nontolerant plants, hybrid lethality perfectly cosegregates with tolerance [19],[20]. The simplest explanation is that the copper tolerance allele that spread to fixation in the Copperopolis population also happens to cause hybrid lethality as a pleiotropic by-product. The alternative explanation is that the copper tolerance and hybrid lethality loci happen to be genetically linked; when the copper tolerance allele spread to fixation in Copperopolis, hybrid lethality hitchhiked to high frequency along with it [20]. But with 2*n* = 28 chromosomes, the odds that copper tolerance and hybrid lethality alleles happen to be linked would seem vanishingly small [20].

**Figure 1:**
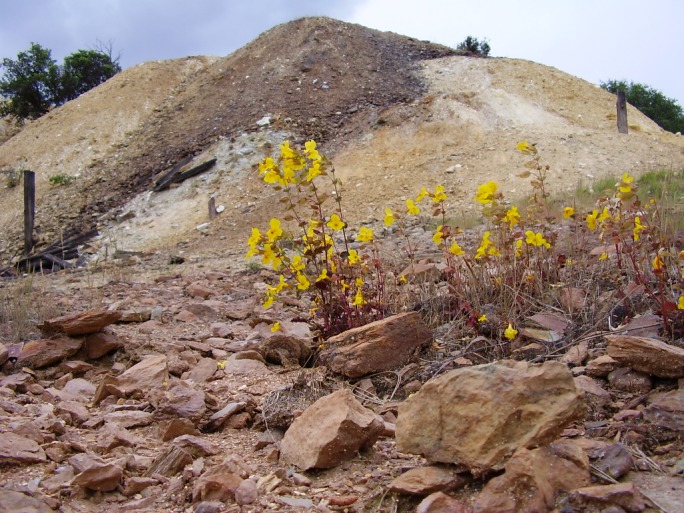
Yellow monkey flowers (*Mimulus guttatus*) growing in the heavy-metal contaminated soils of copper-mine tailings.

In this issue, Wright and colleagues [21] revisit this classic case of genetic incompatibility as a by-product of ecological adaptation. They make two discoveries, one genetic and the other evolutionary. By conducting extensive crossing experiments and leveraging the *M. guttatus* genome sequence (www.mimulusevolution.org), Wright et al. [21] map copper tolerance and hybrid necrosis to tightly linked but genetically separable loci, *Tol1* and *Nec1*, respectively. Hybrid lethality is not a pleiotropic consequence of copper tolerance. Instead, the tolerant *Tol1* allele spread to fixation in Copperopolis, and the tightly linked incompatible *Nec1* allele spread with it by genetic hitchhiking. In a turn of bad luck, the loci happen to fall in a heterochromatic pericentric region, where genome assemblies are often problematic, putting identification of the *Tol1* and *Nec1* genes out of immediate reach. Wright et al. [21] were, however, able to identify linked markers within ∼0.3 cM of *Tol1* and place *Nec1* within a 10-kb genomic interval that contains a *Gypsy3* retrotransposon, raising two possibilities. First, the *Gypsy3* element is unlikely to cause hybrid lethality directly; instead, as transposable elements are often epigenetically silenced in plants, it seems possible that the *Nec1*-associated *Gypsy3* is silenced with incidental consequences for gene expression on a gene (or genes) in the vicinity [22]. Second, although the *Nec1* interval is 10-kb in the reference genome of *M. guttatus*, it could be larger in the (not-yet-sequenced) Copperopolis population, perhaps harboring additional genes.

With *Tol1* and *Nec1* mapped near and to particular genomic scaffolds, respectively, Wright et al. were able to investigate the evolutionary history of the genomic region. Given the clear adaptive significance of copper tolerance in Copperopolis plants, we might expect to see the signatures of a strong selective sweep in the *Tol1* region—a single *Tol1* haplotype may have spread to fixation so quickly that all Copperopolis descendant plants bear the identical haplotype and thus show strongly reduced population genetic variability in the *Tol1-Nec1* region relative to the rest of the genome [23],[24]. After the selective sweep is complete, variability in the region ought to recover gradually as new mutations arise and begin to fill out the mutation-drift equilibrium frequency spectrum expected for neutral variation in the Copperopolis population [25],[26]. Given that *Tol1* reflects an adaptation to mine tailings established just ∼150 generations ago, there would have been little time for such a recovery. And yet, while Wright et al. find evidence of moderately reduced genetic variability in the *Tol1-Nec1* genomic region, the magnitude of the reduction is hardly dramatic relative to the genome average.

How, then, is it possible that the *Tol1-Nec1* region swept to fixation in Copperopolis in fewer than ∼150 generations and yet left no strong footprint of a hitchhiking event? One possibility is that rather than a single, unique *Tol1-Nec1* haplotype contributing to fixation, causing a “hard sweep," *multiple Tol1-Nec1* haplotypes sampled from previously standing genetic variation contributed to fixation, causing a “soft sweep" [27]. A soft sweep would be plausible if *Tol1* and *Nec1* both segregate in the local off-mine ancestral population and if the two were, coincidentally, found on the same chromosome more often than expected by chance (i.e., in linkage disequilibrium). Then, after the copper mines were established, multiple plants with multiple *Tol1* haplotypes (and, by association, *Nec1*) could have colonized the newly contaminated soils of the mine tailings. *Tol1* segregates at ∼9% in surrounding populations, suggesting that standing genetic variation for copper tolerance may well have been present in the ancestral populations.

Two big questions remain for the *Tol1-Nec1* story, and both would be readily advanced by identification of *Tol1* and *Nec1*. The first question concerns the history of *Tol1* haplotypes in Copperopolis and surrounding off-mine populations. As *Nec1*-mediated hybrid lethality is incomplete, the ∼9% *Tol1* frequency in surrounding populations could reflect its export via gene flow from the Copperopolis populations. Conversely, if there was a soft sweep from standing *Tol1* variation in surrounding off-mine populations, then *Tol1* and *Nec1* may still be in linkage disequilibrium in those populations (assuming ∼150 years of recombination has not broken up the association). Resolving these alternative possibilities is a matter of establishing the history of movement of *Tol1* haplotypes into or out of the Copperopolis population. The soft sweep scenario, if correct, presents a population genetics puzzle: during the historical time that mutations accumulated among the multiple tolerant but incompatible *Tol1*-*Nec1* haplotypes in the ancestral off-mine populations, why did recombination fail to degrade the association, giving rise to tolerant but *compatible* haplotypes?

The second question concerns the identity of *Nec1* (or if it really is a *Gypsy3* element, the identity of the nearby gene whose expression is disrupted as a consequence). The answer bears on one of the new emerging generalizations about genetic incompatibilities in plants [9]. Recently, Bomblies and Weigel [28] synthesized a century's worth of observations on the commonly seen necrosis phenotype in plant hybrids and, based on their own genetic analyses in *Arabidopsis* [29], suggested that many of these cases may have a common underlying basis: incompatibilities between plant pathogen resistance genes can cause autoimmune responses that result in tissue necrosis and hybrid lethality. Hybrid necrosis, indeed, appears to involve pathogen resistance genes across multiple plants groups [9],[28]. It remains to be seen if the *Nec1*-mediated lethality provides yet another instance.
